# Supplementary material for: Biodistribution of Nanostructured Lipid Carriers in Mice Atherosclerotic Model
Source: Molecules. 2019 Sep 26;24(19):3499. doi: 10.3390/molecules24193499 (PMC6803849; doi:10.3390/molecules24193499)
Supplement: Supplementary file 1 [file molecules-24-03499-s001.pdf]

Biodistribution of nanostructured lipid carriers in mice atherosclerotic model: Supplementary Information

Laurent Devel [1], Gunter Almer [2], Claudia Cabella [3], Fabrice Beau [1], Mylène Bernes [1], Paolo Oliva [3], Fabrice Navarro [4], Ruth Prassl [5], Harald Mangge [2], and Isabelle Texier [4,*]

[1] CEA, Institut des Sciences du Vivant Frédéric Joliot, Service d'Ingénierie Moléculaire des Protéines (SIMOPRO), Université Paris-Saclay, Gif-sur-Yvette 91190, France ; laurent.devel@cea.fr
[2] Clinical Institute of Medical and Chemical Laboratory Diagnostics, Medical University of Graz, Austria; gunter.almer@medunigraz.at
[3] Centro Ricerche Bracco, Bracco Imaging SpA, Colleretto Giacosa, Italy; claudia.cabella@bracco.com
[4] University Grenoble Alpes, CEA, LETI, F-38000 Grenoble, France; isabelle.texier-nogues@cea.fr
[5] Institute of Biophysics, Medical University of Graz, Austria; ruth.prassl@medunigraz.at
* Correspondence: Isabelle.texier-nogues@cea.fr; Tel.: +33-438-784-670 (I.T.)

General information

BocNH-PEG$_{100}$-NH$_2$ was purchased from Iris Biotech GmbH (Marktredwitz, Germany), succinimidyl 3-(2-pyridyldithio)propionate (SPDP) and AMAS (N-α-maleimidoacet-oxysuccinimide ester) were purchased from Thermo Fisher Scientific (Les Ulis, France). Phosphinic building block **A** (see scheme S2 for structure) was synthesized as previously described [1]. All other chemicals were purchased from Sigma-Aldrich (Saint-Quentin Fallavier, France). They were analytical grade and used without further purification unless otherwise specified. Universal polyethylene glycol (PEG) NovaTagTM resin (code: 855058, substitution: 0.24 mmol/g) and Fmoc-(L)-Glu(OtBu)-OH (code: 852009) were purchased from Merck Millipore (Darmstadt, Germany). Pseudo peptide synthesis was performed manually in polypropylene syringe equipped with a polyethylene frit and a stopper. Microwave experiments were performed on a Discover apparatus (CEM µWave, Matthews, NC, USA) using open vessel mode with SPS kit. Compounds analysis by Reverse Phase High Pressure Liquid Chromatography (RP-HPLC) was performed on an Agilent XDB C18 column (150 × 4.6 mm, 5 µm, flow rate = 0.6 mL/min). Compounds purification was performed on a Supelco Ascentis® C18 column (150 × 10 mm, 5 µm, flow rate = 3 mL/min). UV detection was performed at 230 nm. A solvent system consisting of (A) 0.1% TFA in water and (B) 0.09% TFA in acetonitrile was used. Retention times (Rt) are reported in minutes. UV absorption was performed on a UV-1800 spectrophotometer (Shimadzu, Kyoto Japan). Mass spectrometry data were collected using a 4800 MALDI-TOF mass spectrometer (Applied Biosystems, Foster City, CA) or an ESI ion trap Esquire HCT spectrometer (Bruker Daltonics, Billerica, MA). Amino acid composition was characterized using an aminoTac JLC-500/V amino acid analyzer (JEOL, Tokyo, Japan). Extinction coefficients were calculated from absorbance measurements and solution concentrations determined by amino acid compositions. ^{1}H NMR spectra were measured on a Bruker 300 spectrometer in deuterated chloroform (CDCl$_3$). Peaks were referenced against residual solvent at 7.26 ppm (CDCl$_3$).

Synthesis of surfactant SA-PEG$_{100}$-S-SPyr (1)

The surfactant SA-PEG$_{100}$-S-S-Pyr (**1**) was synthesized according to Scheme S1.

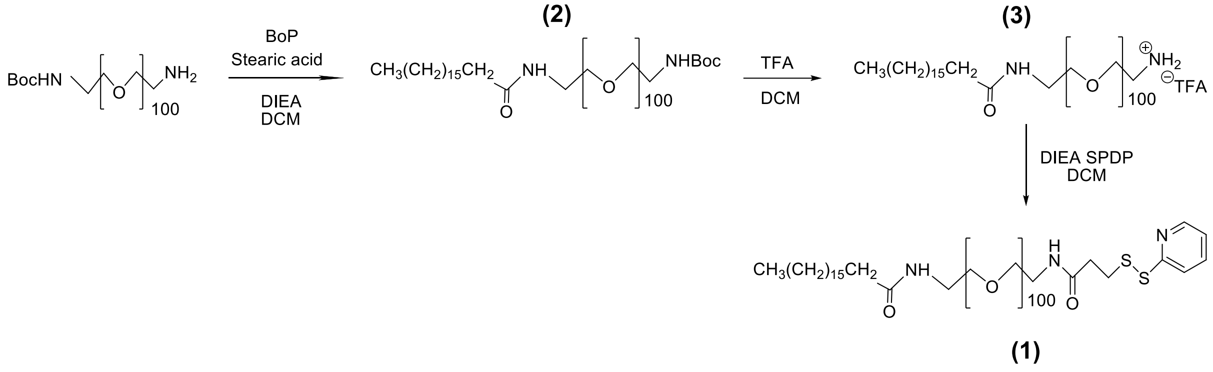

Scheme S1: Synthesis of surfactant SA-PEG_{100}-S-S-Pyr (**1**).

*Synthesis of SA-PEG_{100}-NHBoc (**2**)*

An amount of 1.03 g (3.6 mmol) of stearic acid and 1.6 g (3.6 mmol) of (benzotriazol-1-yloxy)tris(dimethylamino)phosphonium hexafluorophosphate (BOP) were added to 80 mL of dichloromethane until complete dissolution. 5 g of BocNH-PEG_{100}-NH_2 (1.03 mmol) and 470 mg of N,N-Diisopropylethylamine (DIEA) (3.6 mmol) were then added and the mixture was stirred for two hours at room temperature. Solvent was then reduced under reduced pressure and precipitation was obtained upon addition of cold diethyl ether. The precipitate was filtered, dissolved in water, extensively dialysed against water (MWCO 1 kDa) and lyophilised, yielding 3.15 g (0.63 mmol, 61%) of SA-PEG_{100}-NHBoc (**2**).

RMN ^1H (300 MHz ; $CDCl_3$; ppm) : δ : 0.87 (t ; J=7.2 Hz ; 3H) ; 1.24 (m ; 28H) ; 1.44 (s ; 9H) ; 1.67 (quin ; 2H) ; 2.42 (t ; *J*=7.5 Hz ; 2H) ; 3.3 (t ; J=5.0 Hz ; 2H) ; 3.4 (t ; J=5.0 Hz ; 2H) ; 3.48-3.8 (m ; 360H) ; 3.87 (t ; *J*=5.0 Hz ; 2H) ; 7.79 (bs ; 1H).

*Synthesis of SA-PEG_{100}-$NH_3^+TFA^-$ (**3**)*

An amount of 1.61 g (0.3 mmol) of SA-PEG_{100}-NHBoc (**2**) and 6.32 g (55 mmol) of trifluoroacetic acid (TFA) were added to 10 mL of dichloromethane until complete dissolution. The mixture was stirred for one hour at room temperature. Solvent and TFA were then evaporated by co-evaporation with toluene under reduced pressure. Precipitation was obtained upon addition of cold diethyl ether. The precipitate was filtered, dissolved in water, extensively dialysed against water (MWCO 1 kDa) and lyophilised, yielding 1.37 g (0.26 mmol, 85%) of SA-PEG_{100}-$NH_3^+TFA^-$ (**3**).

RMN ^1H (300 MHz ; $CDCl_3$; ppm) : δ : 0.87 (t ; *J*=7.2 Hz ; 3H) ; 1.24 (m ; 28H) ; 1.60 (quin ; 2H) ; 2.15 (t ; *J*=7.5 Hz ; 2H) ; 3.17 (bt ; 2H) ; 3.4 (m ; 4H) ; 3.48-3.8 (m ; 360H) ; 3.87 (t ; *J*=5 Hz ; 2H) ; 6.14 (bs ; 1H) ; 7.9 (bs ; 2H).

*Synthesis of SA-PEG_{100}-S-S-Pyr (**1**)*

An amount of 1.47 g (0.3 mmol) of SA-PEG_{100}-$NH_3^+TFA^-$ (**3**) and 110 mg of DIEA (0.85 mmol) were added to 20 mL of dichloromethane until complete dissolution. 265 mg of SPDP (0.85 mmol) were then added and the mixture was stirred for two hours at room temperature. Solvent was then reduced under reduced pressure and precipitation was obtained upon addition of cold diethyl ether. The precipitate

was filtered, dissolved in water, extensively dialysed against water (MWCO 1 kDa) and lyophilised, yielding 956 mg (0.18 mmol, 61%) of SA-PEG$_{100}$-SPDP (**1**).

RMN ^{1}H (300 MHz ; $CDCl_3$; ppm) : δ : 0.88 (t ; *J*=7.2 Hz ; 2H) ; 1.25 (m ; 28H) ; 1.63 (quin ; 2H) ; 2.17 (t ; *J*=7.5 Hz ; 2H) ; 2.62 (t ; *J*=7 Hz ; 2H) ; 3.09 (t ; *J*=7 Hz ; 2H) ; 3.42 (m ; 4H) ; 3.48-3,8 (m ; 360H) ; 3.88 (t ; *J*=5 Hz ; 2H) ; 6.11 (bt ; 1H) ; 6.79 (bt ; 1H) ; 7.11 (m ; 1H) ; 7.67 (m ; 2H) ; 8.49 (m ; 1H).

Synthesis of RXP470.1-PEG$_2$-NH$_2$-Mal (4)

This derivative was synthesized according to Scheme S2.

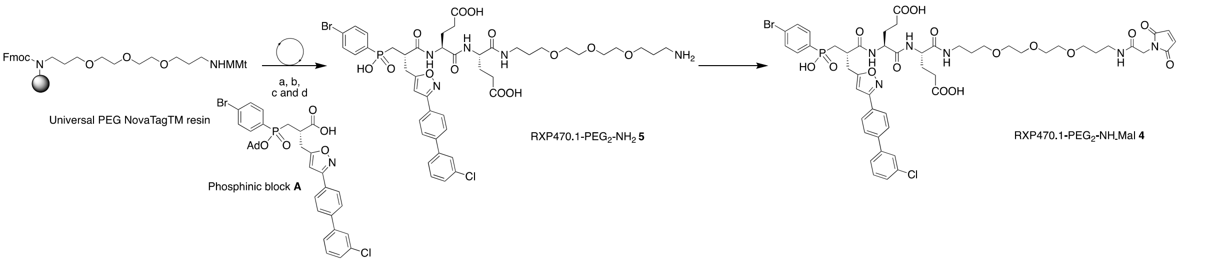

Reagents and conditions: (a) Fmoc solid phase synthesis, Fmoc-Glu(OtBu)-OH (10 equiv), ClHOBt/DIC (10 equiv), DMF; 60°C, microwave irradiation at 45W, 10min, (b) Phosphinic block (1.2 equiv), ClHOBt/DIC (3 equiv), DMF; 60°C, microwave irradiation at 45W, 60min, (c) TFA/TIS/H_2O: 95/2.5/2.5, RT, 3x45 min, (d) RP-HPLC separation, 17% (e) N-α-maleimidoacet-oxysuccinimide ester (1.2 equiv), DIEA (10 equiv), DMF, RT, ON followed by RP-HPLC purification, 30%.

Scheme S2: Synthetic pathway to access RXP470.1-PEG$_2$-NH-Mal (**4**).

Briefly, standard Fmoc methodology was used to build amino acid sequence on Universal PEG NovaTagTM resin (300 mg, 0.074 mmol). Fmoc-Glu(OtBu)-OH (0.315 g, 0.74 mmol, 10 equiv) and phosphinic building block **A** [1] (0.062 g, 0.089 mmol, 1.2 equiv) were incorporated on solid support following a standard protocol in presence of N,N'-diisopropylcarbodiimide (DIC, 10 equiv) and 6-chloro-1-hydroxybenzotriazole di-hydrate (ClHOBt, 10 equiv) in dimethylformamide (DMF). The coupling reactions for Fmoc-Glu(OtBu)-OH amino acid and phosphinic building blocks were performed under microwave irradiations (45 W) at 60°C for 10 min and 60 min, respectively. Amino derivative RXP470-PEG$_2$-NH$_2$ was then cleaved from the support with trifluoroacetic acid/triisopropylsilane/water 95:2.5:2.5 cocktail (5 mL, 3 × 45 min) and with trifluoroacetic acid/dichloromethane solution 1:1 (5 mL, 2 × 45 min). The crude pseudo peptide as a diastereomers mixture was purified by RP-HPLC on a Supelco Ascentis® C18 column (0 to 100% B in 30 min) to lead to compound RXP470.1-PEG$_2$-NH$_2$ **5** (0.0088 g, 17%) as a white powder after freeze drying [1]. To a solution of **5** (0.0029 g, 0.0028 mmol, 1 eq) in DMF (C = 56 mM) were added successively N,N-diisopropylethylamine (4.7 μL, 0.028 mmol, 10 equiv) and a solution of AMAS (0.009 g, 0.0034 mmol, 1.2 equiv). The resulting solution was stirred at room temperature and the progress of reaction was monitored by RP-HPLC (Agilent XDB C18 column, 0 to 100% B in 30 min). The reaction mixture was quenched with water and the crude material was purified by RP-HPLC (Supelco Ascentis® C18 column, 0->3 min: 0 to 40% B, 3->28 min: 40 to 70% B, 28->30 min: 100% B). RXP470.1-PEG$_2$-NH$_2$-Mal **4** was obtained as a white solid after freeze drying (30% yield) and stored at -20°C away from light.

RXP470.1-PEG$_2$-NH$_2$-Mal **4**: In A/B:1/1: ε274 nm = 33,600 M^{-1}. cm^{-1}, Rt = 25.8 min (Supelco Ascentis® Express C18 column, 0 to 100% B in 30 min); MS (ESI, positive mode) m/z for $[C_{51}H_{60}BrClN_6O_{16}P]^+$ calcd 1159.4, found 1159.3. HRMS (MALDI TOF) m/z for $[C_{51}H_{60}BrClN_6O_{16}P]^+$ calcd: 1157,2670, found 1157.3188.

Colloidal stability of NLC and RXP470-NLC

The colloidal stability of NLC and RXP-NLC was assessed by DLS measurements performed for 90 days after particle formulation in 1X PBS (Figure S1).

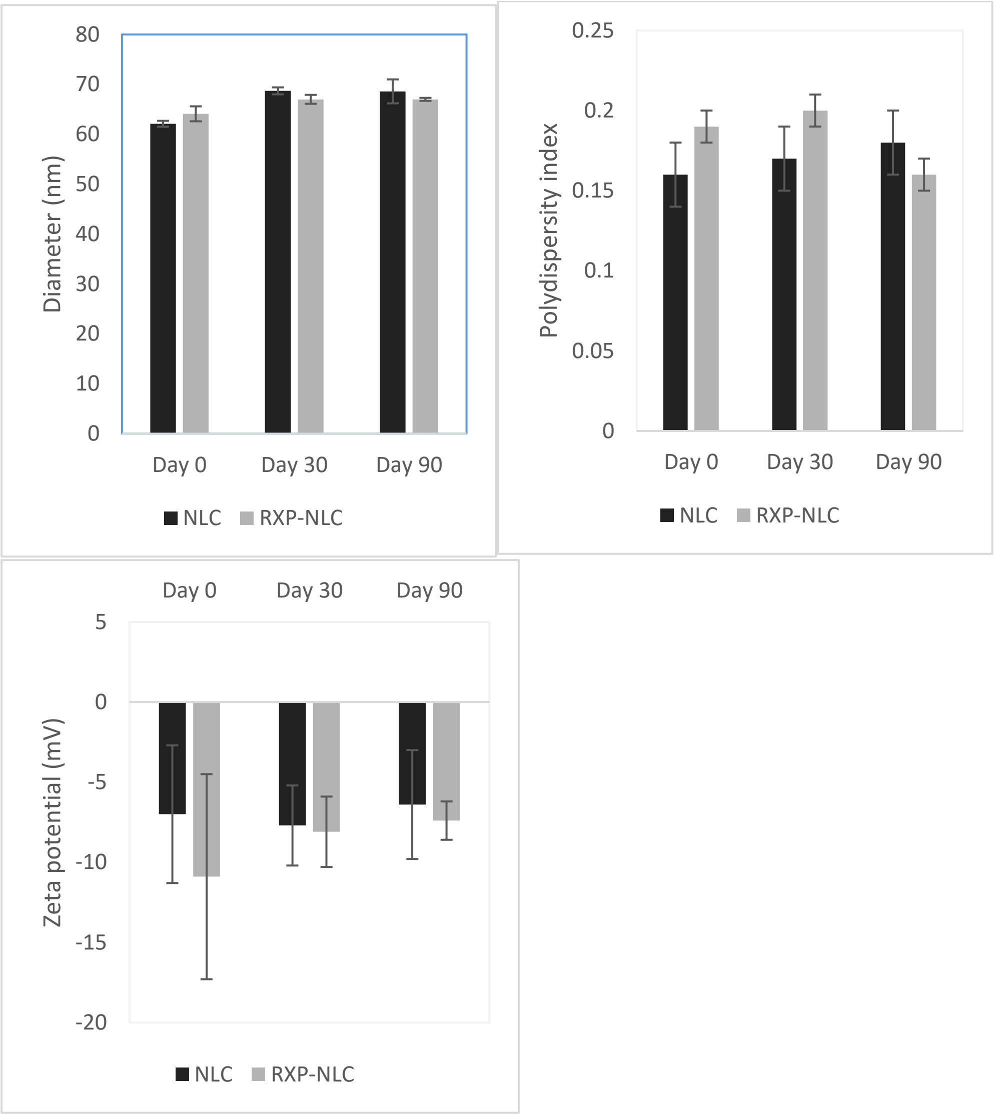

Figure S1: 3-months DLS data of NLC and RXP-NLC.

SEC analysis after RXP470.1 grafting onto nanoparticles

Alternatively to dialysis, RXP-NLC could also be purified by SEC on Sephadex 50 gel phase using 1X PBS for elution. DiD-labelled particles eluted in the 1-4.5 mL fractions, as assessed by fluorescence quantification (Tecan fluorescence microplate reader), whereas unbound RXP470 eluted in 7-17 mL fractions (HPLC quantification) (Figure S2).

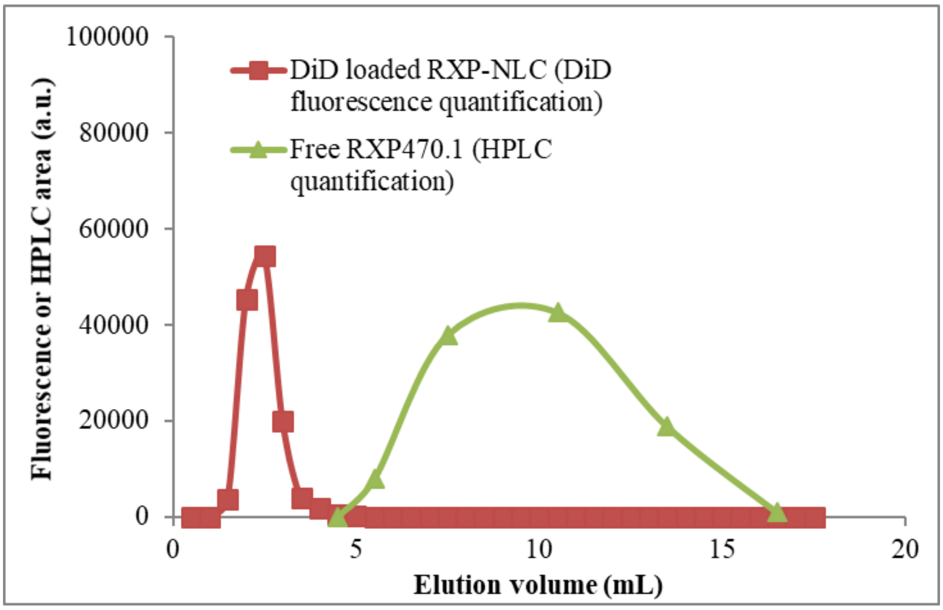

Figure S2: SEC elution profile of RXP-NLC, showing a good separation between nanoparticle fractions (1-4.5 mL) and free RXP470.1 fractions (7-17 mL). (a.u.: arbitrary units).

The comparison of SEC/HPLC profiles obtained after incubation of RXP470.1-maleimide with 1) PBS buffer (reflecting the total quantity of RXP470.1 introduced for the coupling reaction) and 2) NLC-SH suspended in PBS buffer (reflecting the remaining quantity of RXP470.1 not grafted to the nanoparticles) can be used to estimate the quantity of RXP470.1-maleimide grafted onto NLC. The area under the elution curve in case 2) was 51% of that of elution curve in case 1) (Figure S3), resulting in 49% yield for coupling reaction. This indirect quantitation was far less precise than the direct quantitation based on the dosage of glutamate residues described in main text, but provided additional evidence of covalent coupling of RXP470.1 onto NLC.

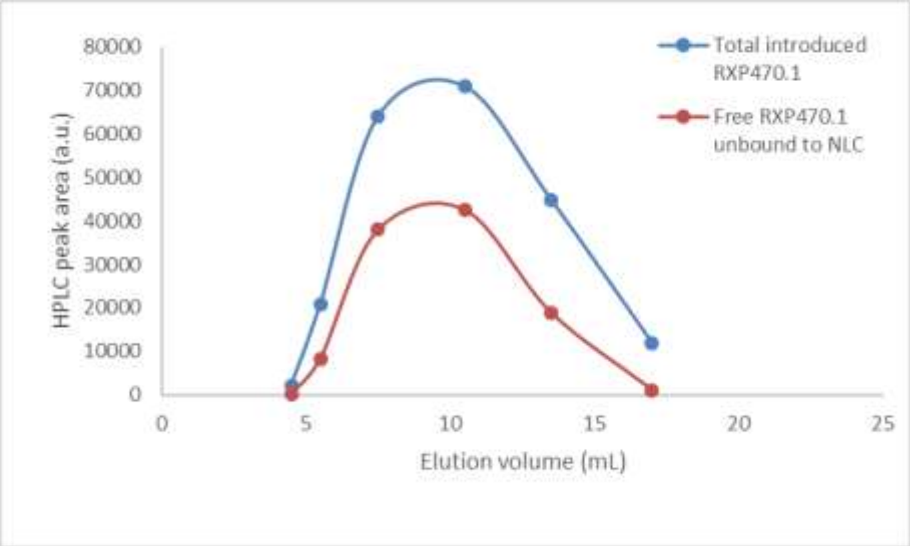

Figure S3: Indirect estimation of NLC-bound RXP470.1 by SEC/HPLC analysis. SEC/HPLC elution profiles of RXP470.1-maleimide incubated with 1) PBS buffer (reflecting the total quantity of RXP470.1 introduced for the coupling reaction) and 2) NLC-SH suspended in PBS buffer (reflecting the remaining quantity of RXP470.1 not grafted to the nanoparticles). (a.u.: arbitrary units).

Organ histology fluorescence microscopy images

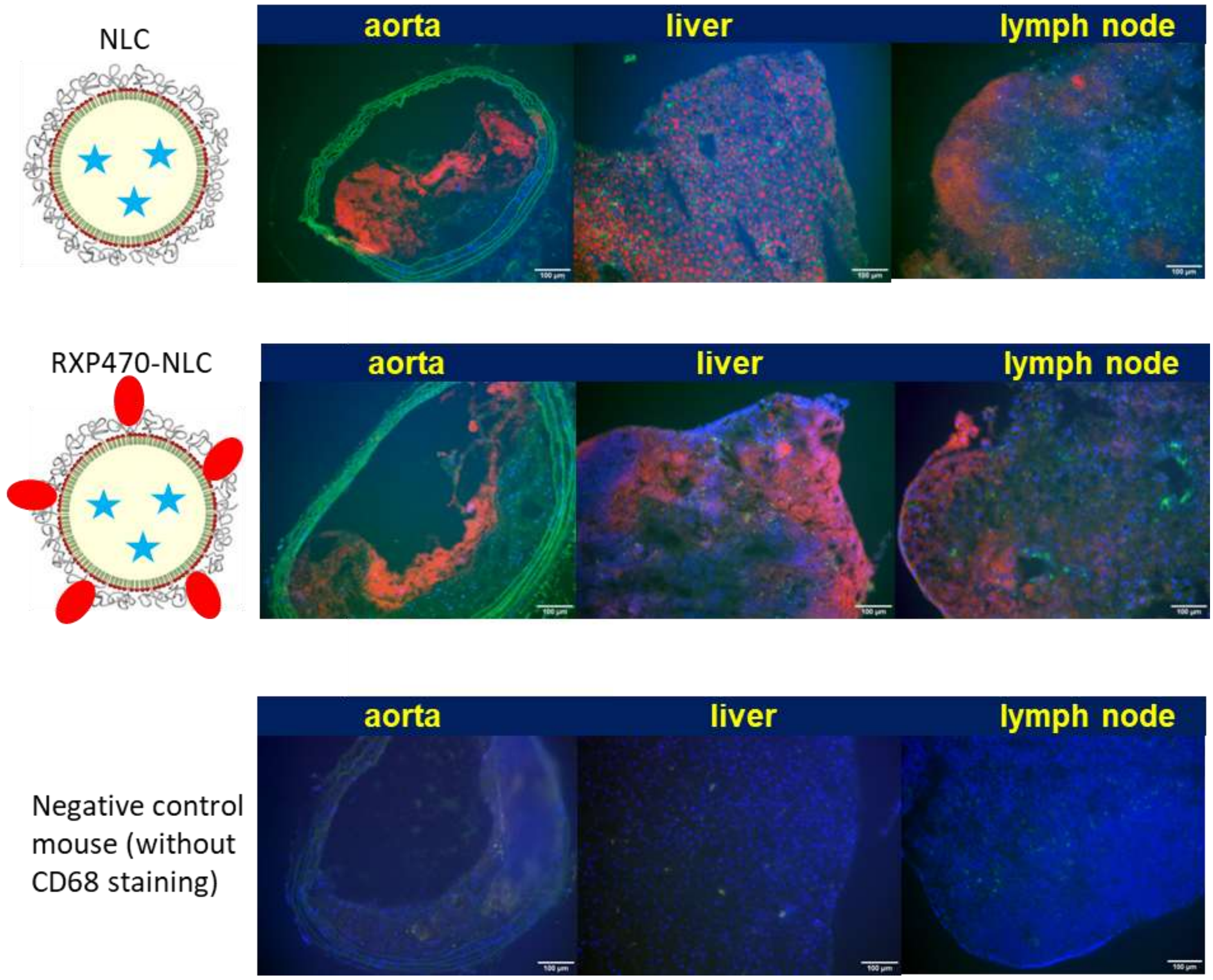

Figure S4: Organ and aorta cross-section histology (fluorescence microscopy). Blue: nucleus (Hoechst), Green: anti-CD68 Ab (macrophage marker) and tissue auto-fluorescence, Red: DiD-loaded NLC. Scale bar: 100 µm.

References

1. Bordenave, Thomas; Helle, Marion; Beau, Fabrice; Georgiadis, Dimitris; Tepshi, Livia; Bernes, Mylène; Ye, Yunpeng; Levenez, Laure; Poquet, Enora; Nozach, Hervé; Razavian, Mahmoud; Toczek, Jakub; Stura, Enrico A.; Dive, Vincent; Sadeghi, Mehran M.; Devel, Laurent. Synthesis and in Vitro and in Vivo Evaluation of MMP-12 Selective Optical Probes. *Bioconjugate Chemistry* **2016**, 27, 2407-2417.
